# Supplementary material for: Structural and Biochemical Analysis of the Dual Inhibition of MG-132 against SARS-CoV-2 Main Protease (Mpro/3CLpro) and Human Cathepsin-L
Source: Int J Mol Sci. 2021 Oct 29;22(21):11779. doi: 10.3390/ijms222111779 (PMC8583849; doi:10.3390/ijms222111779)
Supplement: Supplementary file 1 [file ijms-22-11779-s001.zip › ijms-1421293-SI.pdf]

Structural and biochemical analysis of the dual inhibition of MG-132 against SARS-CoV-2 Main protease (Mpro/3CLpro) and human Cathepsin-L

Author List

Elisa Costanzi[1], Maria Kuzikov[2,3], Francesca Esposito[4], Simone Albani[5,6], Nicola Demitri[1], Barbara Giabbai[1], Marianna Camasta[4], Enzo Tramontano[4], Giulia Rossetti[5,7,8], Andrea Zaliani[2], and Paola Storici[1*]

Affiliations

[1]Elettra - Sincrotrone Trieste, 34149 Trieste, Italy

[2]Fraunhofer Institute for Translational Medicine and Pharmacology (ITMP), 22525 Hamburg, Germany

[3] Jacobs University Bremen gGmbH, 28759 Bremen, Germany

[4]Department of Life and Environmental Sciences, University of Cagliari, 09124 Cagliari, Italy

[5]Institute for Neuroscience and Medicine (INM-9) and Institute for Advanced Simulations (IAS-5) "Computational biomedicine", Forschungszentrum Jülich, 52425 Jülich, Germany.

[6]Department of Biology, Faculty of Mathematics, Computer Science and Natural Sciences, RWTH Aachen, 52062 Aachen, Germany

[7]Jülich Supercomputing Centre (JSC) Forschungszentrum Jülich, D-52425 Jülich, Germany

[8]Department of Neurology, Faculty of Medicine, RWTH Aachen University, 52074 Aachen, Germany

[*]Email: paola.storici@elettra.eu

Supporting Information

Contents

Figure S1: Superposition of apo structures

Figure S2: Overall structures of Mpro bound to MG-132 ± DTT

Figure S3: 2Fo-Fc density contoured at 1σ ofMG-132 observed in the five solved structures.

Figure S4: Superposition of MG-132 bound structures in spacegroup *P* $2_12_12_1$ (teal), *P* 1 (lightblue) and *C* 2 (grey). (A) overall superposition. (B) MG-132 poses in the active sites

FigureS5: Crystal contacts and active site accessibility

Figure S6: (A) Surface representation of the Mpro active site with highlighted the sub-pockets, (B) 2Fo-Fc density contoured at 1σ of the main residues in the active site and the conserved catalytic water

Figure S7: MG-132 activity on SARS-CoV-2 MPro in the presence/absence of DTT

Figure S8: Increase of thermal stability of Mpro at increasing concentration of MG-132

Table S1: Data collection and refinement statistics. Statistics for the highest-resolution shell are shown in parentheses

Table S2: The so-far reported structures of cathepsin L are reported and compared with the *Carica papaya* papain protease (RMSD values).

Figure S1

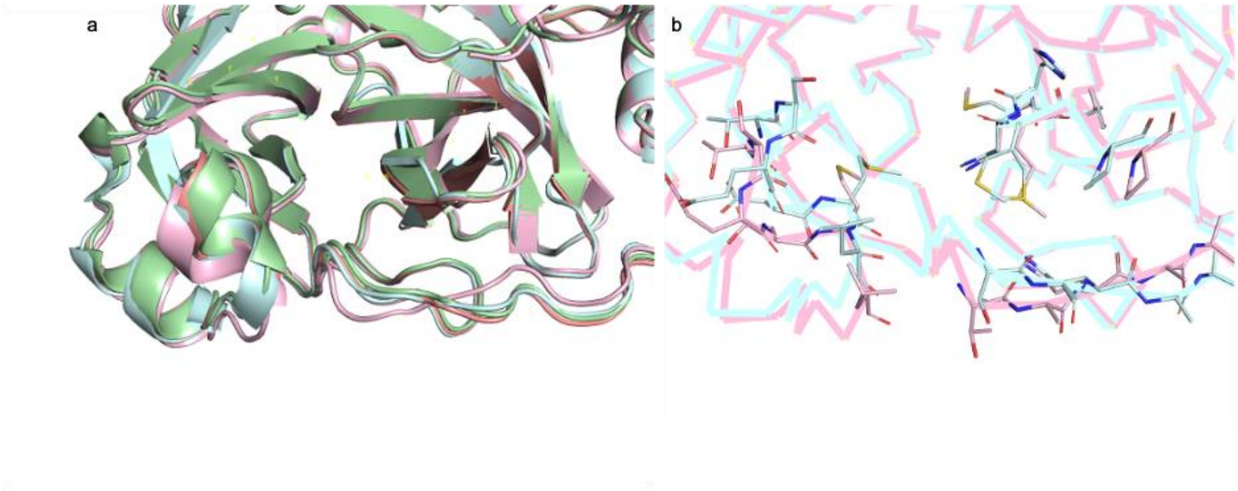

Figure S1: Superposition of apo structures (A) spacegroups *P* $2_12_12_1$ colored in pink, *C* 2 in salmon, *P* 2_1 in light green, *C* 2 at RT in cyan. (B) comparison of the apo structure in spacegroup *P* $2_12_12_1$ with the RT structure (PDB ID: 6WQF).

FIGURE S2

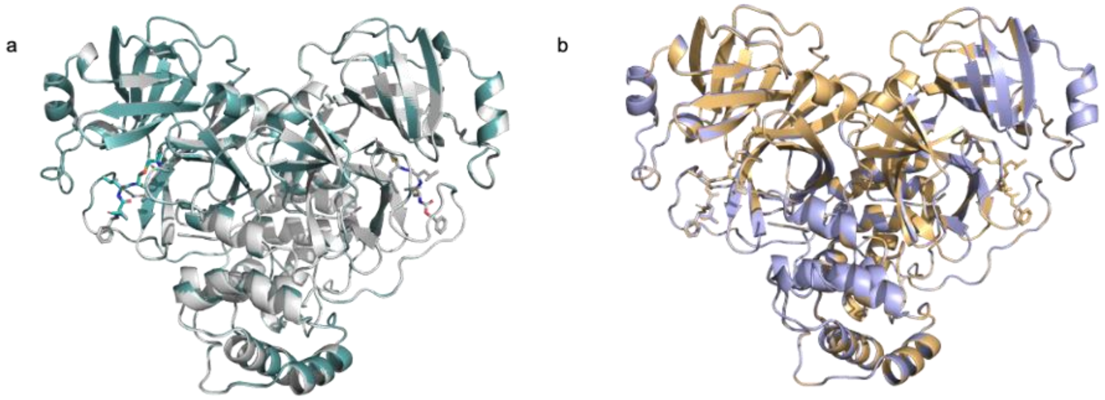

Figure S2: overall structures of Mpro bound to MG-132 ±DTT. (A) superposition of Mpro:MG-132 bound structures in spacegroup *P* $2_12_12_1$ in presence (teal) and absence (light grey) of DTT; (B) superposition of Mpro:MG-132 bound structures in spacegroup *P* 1 in presence (light blue) and absence (light orange) of DTT.

Figure S3

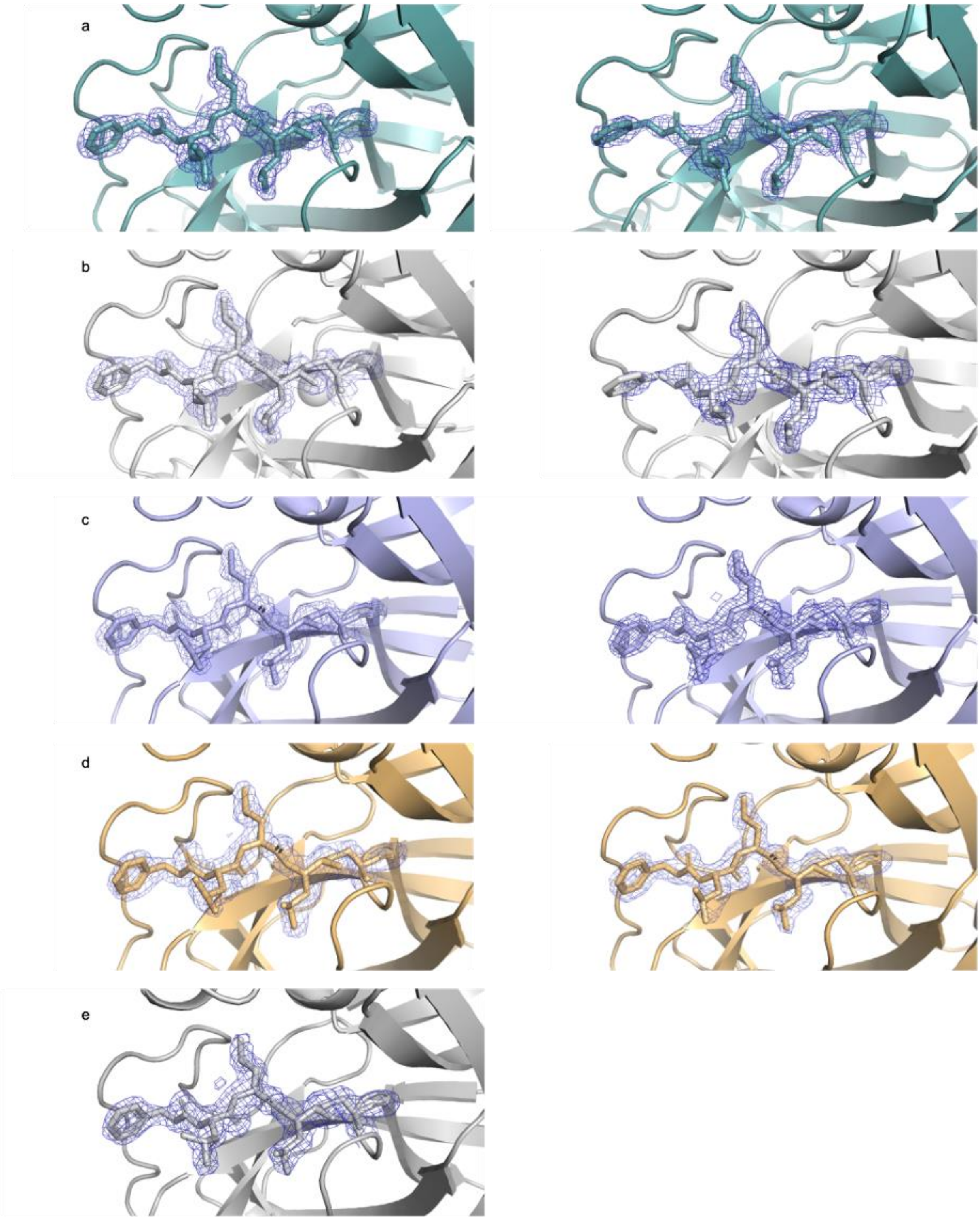

Figure S3: 2Fo-Fc density contoured at 1σ ofMG-132 observed in the five solved structures. (A) spacegroup *P* $2_12_12_1$ in presence of DTT, chain A (left) chain B (right), teal. (B) spacegroup *P* $2_12_12_1$ in absence of DTT, chain A (left) chain B (right), light grey. (C) spacegroup *P* 1 in presence of DTT, chain A (left) chain B (right), light blue. (D) spacegroup *P* 1 in absence of DTT, chain A (left) chain B (right), light orange. (E) spacegroup *C* 2, grey.

Figure S4

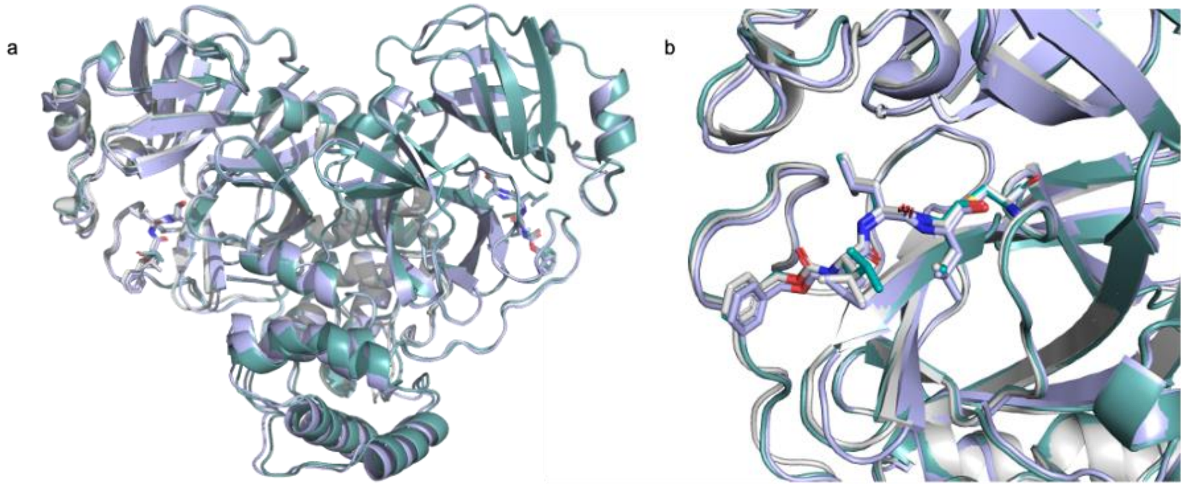

Figure S4: superposition of MG-132 bound structures in spacegroup *P* $2_12_12_1$ (teal), *P* 1 (lightblue) and *C* 2 (grey). (A) overall superposition. (B) MG-132 poses in the active sites.

Figure S5

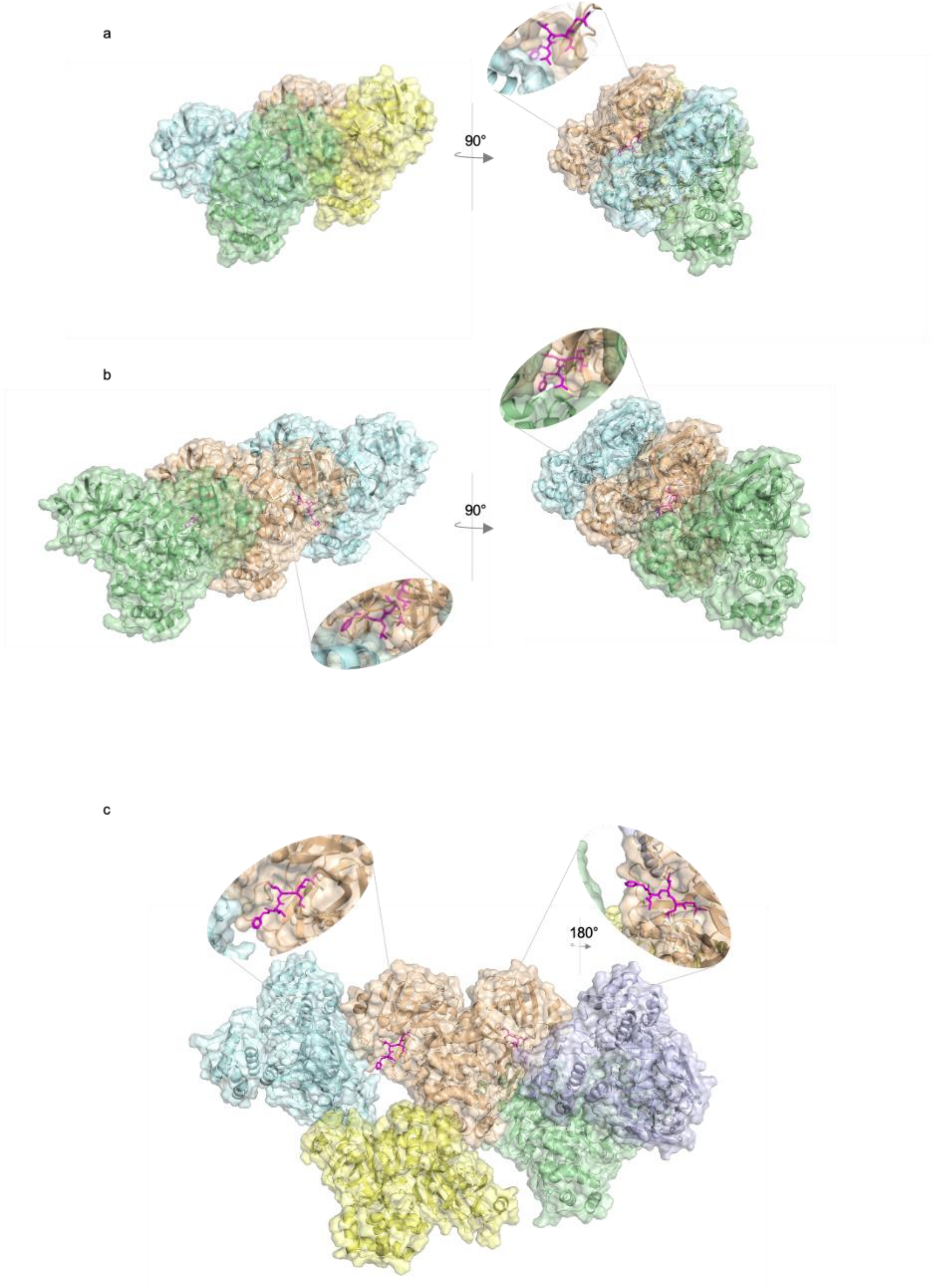

Figure S5: crystal contacts and active site accessibility. Different colors indicate symmetric molecules in the different crystal packings. MG-132 is represented by sticks colored in magenta. (A) spacegroup *C* 2; (B) spacegroup *P* 1; (C) spacegroup $P\ 2_12_12_1$.

Figure S6

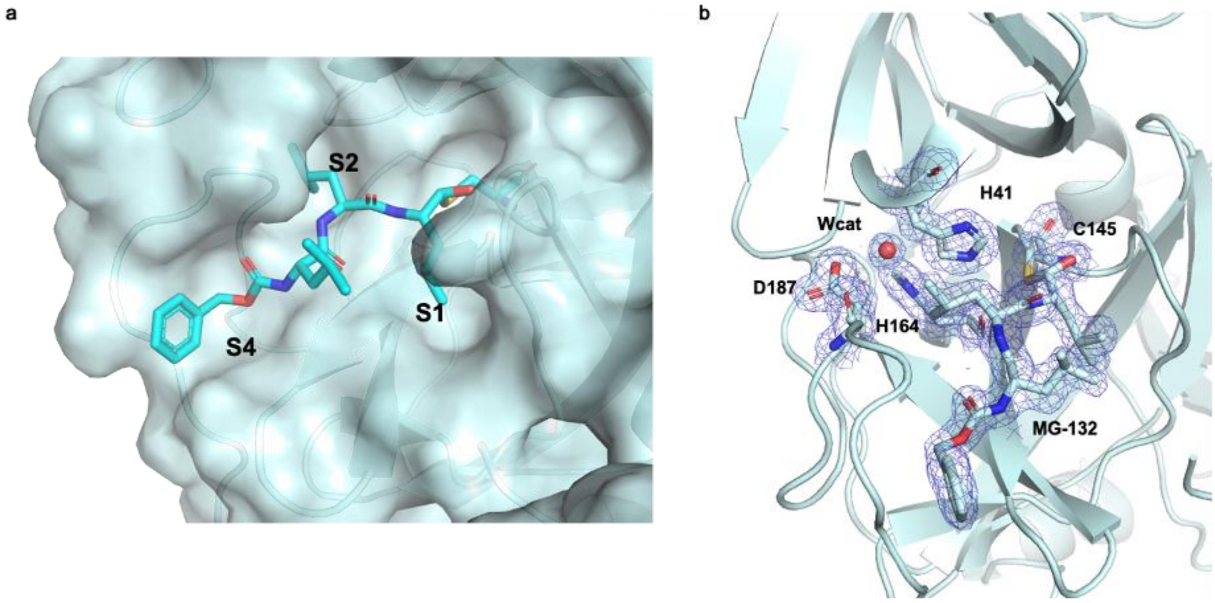

Figure S6: (A) Surface representation of the Mpro active site with highlighted the sub-pockets, (B) 2Fo-Fc density contoured at 1σ of the main residues in the active site and the conserved catalytic water.

Figure S7

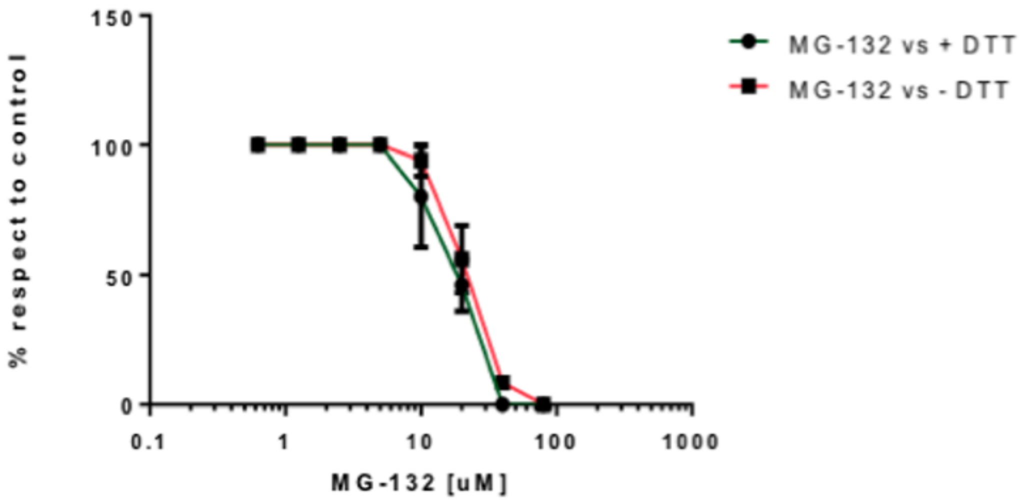

Figure S7: MG-132 activity on SARS-CoV-2 Mpro in the presence/absence of DTT. Dose response curve determined ± 1 mM DTT. The IC_{50} values were 14 and 18.4 uM in the presence/absence of DTT, respectively.

Figure S8

	Tm	St. Dev.	ΔTm
MG-132 1X	53,0	0	0,5
APO + 3% DMSO	51,67	0,29	
MG-132 3X	52,5	0	0,8
APO + 1% DMSO	52,5	0	
MG-132 10X	54,5	0,5	2,0
APO + 2% DMSO	52	0	
MG-132 20X	54,5	0	2,5
APO + 5% DMSO	51,5	0	
MG-132 50X	55,0	0,5	3,5

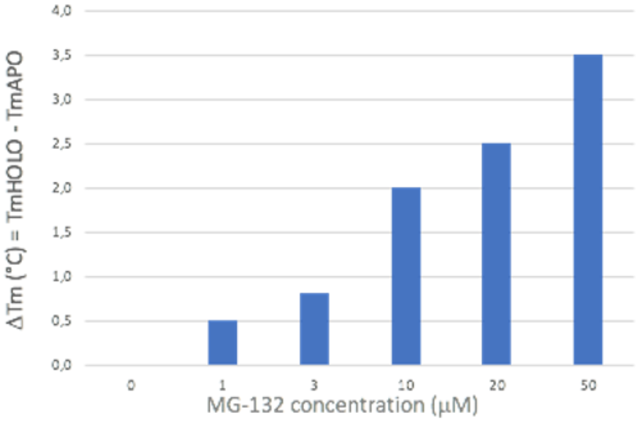

Figure S8: Increase of thermal stability of Mpro with increase of MG-132. Left table reports Tm values for Mpro apo in presence of different concentrations of DMSO (APO) and of Mpro upon addition of increasing amounts of MG-132 that was dissolved in DMSO (HOLO). In the right panel is reported the difference of Tm between MG-132 bound Mpro (HOLO) and the protein apo measured at the corresponding percentage of DMSO. The Mpro concentration in assay was 1 uM.

Table S1: Data collection and refinement statistics of the Mpro apo and inhibitor bound structures solved in different spacegroups and in presence and absence of DTT. Statistics for the highest-resolution shell are shown in parentheses.

	**APO SARS-CoV-2 Mpro**			**MG-132 covalently bonded to SARS-CoV-2 Mpro**				
	C 2	*P* 2_1	*P* $2_12_12_1$	*P* $2_12_12_1$ *w DTT*	*P*$2_12_12_1$ *w/o DTT*	*P 1 w DTT*	*P 1 w/o DTT*	*C 2*
**Data collection**								
PDB ID	7ALH	7ALI	7BB2	7BE7	7BGP	7NG3	7NG6	7NF5
Space group	*C* 2	*P* 2_1	*P* $2_12_12_1$	*P* $2_12_12_1$	*P* $2_12_12_1$	*P* 1	*P* 1	*C* 2
Unit Cell Parameters								
a, *b*, *c* (Å)	115.25, 54.21, 44.80	44.78, 53.70, 114.55	67.52, 100.03, 103.75	67.898, 99.24, 103.375	67.853, 99.779, 103.514	47.28, 54.92, 59.50	47.08, 54.67, 59.33	110.02, 55.08, 47.5[illegible]
α, β, γ (°)	90, 101.18, 90	90, 101.17, 90	90, 90, 90	90, 90, 90	90, 90, 90	114.65, 99.96, 90.54	114.36, 99.82, 90.88	90, 101.23, 90
Cell volume (Å^3)	274254	270203	700705	696561	700821	137741	136414	282464
Solvent content (%)	39.32	38.41	52.5	52.22	52.51	39.59	39.55	41.08
Matthews coefficient (Å^3/Da)	2.03	2.00	2.59	2.57	2.59	2.04	2.03	2.09
Wavelength (Å)	1.000	1.000	0.9716	1.000	1.000	0.999	0.999	0.999
Resolution (Å)	56.52-1.65 (1.68-1.65)	48.45-1.65 (1.68-1.65)	103.75-1.60 (1.63-1.60)	99.24-1.68 (1.71-1.68)	56.11-1.68 (1.71-1.68)	53.05-1.80 (1.84-1.80)	53.01-1.87 (1.91-1.87)	53.96-1.94 1.99-1.94
Number of unique reflections	32583 (1602)	63251 (3121)	93301 (4539)	80180 (4066)	80614 (4098)	47488 (2756)	42278 (2622)	20793 (1422)
R_{merge}	0.056 (0.983)	0.069 (1.104)	0.096 (1.947)	0.098 (1.470)	0.092 (1.469)	0.071 (0.827)	0.071 (0.587)	0.146 (1.379)
R_{meas}	0.063 (1.113)	0.078 (1.247)	0.100 (2.028)	0.106 (1.588)	0.100 (1.603)	0.085 (0.996)	0.084 (0.694)	0.159 (1.496)
R_{pim}	0.029 (0.512)	0.036 (0.575)	0.028 (0.563)	0.040 (0.594)	0.039 (0.634)	0.045 (0.545)	0.045 (0.366)	0.062 (0.574)
$\langle I/\sigma(I) \rangle$	12.3 (1.4)	9.8 (1.4)	15.2 (1.5)	11.9 (1.5)	12.5 (1.5)	10.4 (1.5)	8.5 (1.5)	8.0 (1.5)
$CC^{1/2}$	0.999 (0.625)	0.998 (0.657)	0.999 (0.615)	0.999 (0.619)	0.999 (0.604)	0.998 (0.566)	0.998 (0.765)	0.997 (0.637)
Completeness (%)	99.7 (100.0)	98.4 (97.5)	100.0 (100.0)	99.9 (99.9)	99.8 (100.0)	96.6 (95.6)	96.8 (93.8)	100.0 (100.0)
Multiplicity	4.4 (4.5)	4.5 (4.6)	12.7 (12.7)	7.0 (7.0)	6.3 (6.3)	3.4 (3.3)	3.5 (3.5)	6.4 (6.6)
**Refinement**								
Resolution (Å)	43.93-1.65	38.46-1.65	55.96-1.60	45.84-1.68	45.94-1.68	49.71-1.80	49.57-1.87	49.06-1.94
Num. of reflections	32576	63083	93191	80087	80513	47474	42256	20781
Num. of reflections (R-Free)	1603	3142	4635	3972	3988	2412	2113	1033
R_{work}/R_{free} (%)	16.47 / 18.90	18.88 / 21.67	16.42 / 18.86	17.76 / 20.13	17.04 / 20.10	17.87 / 21.47	16.19 / 20.77	18.19 / 22.46
r.m.s. deviations								
Bond length (Å)	0.007	0.005	0.007	0.005	0.010	0.011	0.007	0.013
Bond angles (°)	0.892	0.849	0.973	0.901	1.005	0.944	0.954	1.071
Ramachandran plot								
favored (%)	99.01	98.68	98.43	98.18	98.01	98.01	97.51	98.35
allowed (%)	0.99	1.32	1.57	1.65	1.99	1.66	2.16	1.32
outliers (%)	0.00	0.00	0.00	0.17	0.00	0.33	0.33	0.33

Table S2: Cathepsin-L structures are reported and compared with the *Carica papaya* papain protease (RMSD values). The co-crystalized ligand, when present, is evaluated for its chemical similarity (e.g. Tanimoto index) [1]to MG-132.

PDB id	*RMSD to 1BP4 (Å)*	*Ligand ID*	*Tanimoto Score (MG-132)*
3OF9	0.765	I0X	0.455
3OF8	0.758	I0Y	0.453
3H8C	0.78	NSZ	0.280
3H8B	0.75	NSY	0.262
3BC3	0.721	OPT	0.253
3H89	0.758	NSX	0.247
3HHA	0.725	NOW	0.228
3HWN	0.748	BD3	0.200
6EZX	0.724	C7Q	0.156
2YJ2	0.715	YJ2	0.149
2XU5	0.726	XU5	0.148
2XU4	0.733	DJT	0.148
2XU3	0.782	XU3	0.144
2YJC	0.724	424	0.138
2XU1	0.742	424	0.138
6EZP	0.76	C3E	0.138
2YJ8	0.719	YJ8	0.136
2YJ9	0.716	YJ9	0.132
2YJB	0.747	YJ9	0.132
5MQY	0.738	GH4	0.126
5F02	0.726	5T9	0.115
4AXM	0.736	V65	0.115
6F06	0.768	C7T	0.105
5MAE	0.726	7KN	0.056
5MAJ	0.717	7KH	0.052
5I4H	0.623	no lig	-

1MHW	0.658	no lig	-
6JD0	0.681	no lig	-
1ICF	0.694	no lig	-
1CJL	0.705	no lig	-
6JD8	0.725	no lig	-
4AXL	0.736	no lig	-
3IV2	0.741	no lig	-
3K24	0.751	no lig	-
2VHS	0.757	no lig	-
3KSE	0.767	no lig	-
1CS8	0.771	no lig	-

REFERECES:

(1) Bajusz, D.; Rácz, A.; Héberger, K. Why Is Tanimoto Index an Appropriate Choice for Fingerprint-Based Similarity Calculations? *J. Cheminformatics* **2015**, *7* (1), 20. https://doi.org/10.1186/s13321-015-0069-3.
